# A natural language processing and deep learning approach to identify child abuse from pediatric electronic medical records

**DOI:** 10.1371/journal.pone.0247404

**Published:** 2021-02-26

**Authors:** Akshaya V. Annapragada, Marcella M. Donaruma-Kwoh, Ananth V. Annapragada, Zbigniew A. Starosolski

**Affiliations:** 1 Harvard John A. Paulson School of Engineering and Applied Sciences, Harvard University, Cambridge, MA, United States of America; 2 Pediatrics—Public Health, Baylor College of Medicine, Houston, TX, United States of America; 3 The Singleton Department of Pediatric Radiology, Texas Children’s Hospital, Houston, TX, United States of America; 4 Department of Radiology, Baylor College of Medicine, Houston, TX, United States of America; Taipei Medical University, TAIWAN

## Abstract

Child physical abuse is a leading cause of traumatic injury and death in children. In 2017, child abuse was responsible for 1688 fatalities in the United States, of 3.5 million children referred to Child Protection Services and 674,000 substantiated victims. While large referral hospitals maintain teams trained in Child Abuse Pediatrics, smaller community hospitals often do not have such dedicated resources to evaluate patients for potential abuse. Moreover, identification of abuse has a low margin of error, as false positive identifications lead to unwarranted separations, while false negatives allow dangerous situations to continue. This context makes the consistent detection of and response to abuse difficult, particularly given subtle signs in young, non-verbal patients. Here, we describe the development of artificial intelligence algorithms that use unstructured free-text in the electronic medical record—including notes from physicians, nurses, and social workers—to identify children who are suspected victims of physical abuse. Importantly, only the notes from time of first encounter (e.g.: birth, routine visit, sickness) to the last record before child protection team involvement were used. This allowed us to develop an algorithm using only information available prior to referral to the specialized child protection team. The study was performed in a multi-center referral pediatric hospital on patients screened for abuse within five different locations between 2015 and 2019. Of 1123 patients, 867 records were available after data cleaning and processing, and 55% were abuse-positive as determined by a multi-disciplinary team of clinical professionals. These electronic medical records were encoded with three natural language processing (NLP) algorithms—Bag of Words (BOW), Word Embeddings (WE), and Rules-Based (RB)—and used to train multiple neural network architectures. The BOW and WE encodings utilize the full free-text, while RB selects crucial phrases as identified by physicians. The best architecture was selected by average classification accuracy for the best performing model from each train-test split of a cross-validation experiment. Natural language processing coupled with neural networks detected cases of likely child abuse using only information available to clinicians prior to child protection team referral with average accuracy of 0.90±0.02 and average area under the receiver operator characteristic curve (ROC-AUC) 0.93±0.02 for the best performing Bag of Words models. The best performing rules-based models achieved average accuracy of 0.77±0.04 and average ROC-AUC 0.81±0.05, while a Word Embeddings strategy was severely limited by lack of representative embeddings. Importantly, the best performing model had a false positive rate of 8%, as compared to rates of 20% or higher in previously reported studies. This artificial intelligence approach can help screen patients for whom an abuse concern exists and streamline the identification of patients who may benefit from referral to a child protection team. Furthermore, this approach could be applied to develop computer-aided-diagnosis platforms for the challenging and often intractable problem of reliably identifying pediatric patients suffering from physical abuse.

## 1. Introduction

Child abuse is a leading cause of traumatic injury and death in children [1]. In 2017, Child Protective Services (CPS) received 3.5 million referrals and substantiated concerns of maltreatment in 674,000 children [2]. Child maltreatment was responsible for ~1700 fatalities in the United States, 70% of which were under 3 years of age [2]. Identifying child abuse is critically important for the prevention of escalating injury and death and represents a complex, resource-intensive process, with little room for error.

While large referral hospitals can maintain teams trained in Child Abuse Pediatrics (CAP), smaller community hospitals rarely have such resources, making the consistent detection of and response to subtle signs and symptoms of abuse difficult. Inflicted injury recognition is further complicated by the low margin for error [1]. False positive identifications can lead to separation of a child from appropriate, caring family, while false negatives leave a child in a dangerous situation. Unlike many diagnostic tasks where sensitivity is essential, but low specificity may be overcome by secondary testing, both sensitivity and specificity of abuse detection are crucial.

Current algorithmic approaches for detection of child abuse are sensitive, but compromise specificity. For example, using a clinical decision rule of 4 variables to classify abusive head trauma, sensitivity was 0.96, while specificity was only .46 [3]. Another predictive risk model to aid CPS call screeners respond to allegations of abuse reached area under the receiver operator characteristic curve (ROC-AUC) ~ 0.8, with a false positive rate ~0.2 [4]. Those approaches with increased specificity (0.86) remain limited to single aspects of child maltreatment, such as the clinical prediction of abusive head trauma [5].

Deep learning exhibits potential in diverse clinical tasks ranging from analysis of images to natural language processing (NLP) of electronic medical records (EMR) [6]. For example, deep learning has been used in tasks as diverse as the prediction of drug interactions, analysis of cancer types, classification of radiological images, and stratification of diseased patients [7–9]. The application of NLP to EMR free-text is particularly promising because the EMR is a complete record, including clinical impressions, social and medical history, summaries of diagnostic tests and studies, and longitudinal documentation of the patient’s course. A variety of artificial intelligence (AI) platforms have utilized adult EMRs [10–16] for feature extraction and NLP. However, to our knowledge, only one study utilized NLP of EMRs for abuse identification, and employed conventional machine learning methods rather than the deep learning approaches described here [17].

We developed and evaluated NLP-based AI models utilizing free text from pediatric EMRs to classify suspected child victims into abuse-positive and abuse-negative groups, for potential work up by CAP team physicians. Importantly, we performed NLP using only the notes from time of first encounter (e.g.: birth, routine visit, sickness) to the last record before child protection team involvement. This allowed us to develop classification algorithms using only information available prior to referral to the specialized child protection team. We implemented 3 common NLP encoding techniques, Bag of Words–Term Frequency Inverse Document Frequency (BOW-TFIDF), Word Embeddings (WE) and, Rules-Based (RB), to encode the records, and applied the encodings to train Multi-Layer Perceptron neural networks (MLP). We selected models with both high accuracy and high ROC-AUC. Finally, we compared different encoding techniques and MLP models to assess their strengths and weaknesses.

## 2. Methods

Protocol number: H-44817, was approved by Institutional Review Board for Baylor College of Medicine and Affiliated Hospitals (IRB). Waiver of consent granted by IRB.

### 2.1. Data selection and pre-processing

This retrospective study was conducted under a protocol approved by the Institutional Review Board. All cases investigated by the Child Abuse Pediatrics (CAP) Team between 1/1/2015 and 5/1/2019 were identified. Cases are referred to the CAP Team by clinical departments and social workers within the hospital, county Child Protective Services, and community pediatricians from the hospital’s satellite referral sites at five locations (S1 Fig). The free text notes from every encounter of these patients with staff, as well as radiology reports and summaries of lab tests were selected. All identifying information including names of patients, siblings, and associated adults, addresses, and phone numbers were stripped. For each patient, the note documenting the first encounter with a member of the CAP team was identified and discarded, *along with all subsequent notes*, *ensuring model training and testing using only information available prior to CAP Team evaluation*. This truncation was performed intentionally to replicate the clinical circumstances and available data that referring providers and emergency department clinicians have at their disposal when considering child maltreatment in their differential diagnosis. Records with <2 notes remaining were excluded. All provider notes were merged into a corpus for each patient, and cleaned of extraneous whitespace, single-character words, numbers, punctuation, and the NLTK stop words (frequently occurring but semantically inconsequential English words, per the Natural Language Tool Kit [18]).

### 2.2. Multidimensional representation of data

Three representative NLP techniques (BOW-TFIDF), Word Embeddings (WE), and Rules-Based (RB)) were selected for this study. Each encodes the corpora as numerical matrices. BOW-TFIDF weights words by their number of occurrences in a given corpus relative to all corpora [19]. High weights are given to those words frequent in the corpus being encoded but infrequent overall.

WE uses a pre-trained “embedding” to assign an encoding vector to each word. The embedding is trained on a reference corpus (ideally related to the specific text corpora). The embedding consists of an n-dimensional vector for each word in the reference corpus; vectors representing similar words are close together in n-space. This pre-trained embedding is then used to encode the corpora to be used in the classification task. Words in the corpora that appear in the embedding are assigned the corresponding vector; words that do not appear in the embedding are assigned random vectors. The vectors representing the encoded document can then be used as inputs to a classifier [20, 21]. There is no publicly available text corpus related to pediatric abuse case analysis, therefore two embeddings were selected for this study: the publicly available GloVE embedding (WE-GLOVE, Global Vectors for word representation) utilizing 100 dimensional representations of words trained on the entirety of Wikipedia [22], and an in-house embedding based on the MIMIC-III database (WE-MIMIC, Medical Information Mart for Intensive Care) trained on the notes in the MIMIC-III database, a set of adult ICU records from Beth-Israel-Deaconess Medical Center [23]. We speculated that the MIMIC-III database could yield a more representative embedding than GloVE due to its medical context.

In the RB approach, a CAP team physician selected 88 phrases that were likely to indicate providers’ concerns for abuse. This approach incorporates clinician knowledge, and is similar to that often applied in the biomedical literature where specific features from the medical record are used as classifier inputs rather than all unstructured text [24]. Each patient-level corpus was encoded with these phrases to create a vector of length 88 indicating the application of each rule to the corpus, with 1 indicating the presence of a phrase associated with positive concerns for abuse and 0 indicating the presence of a phrase associated with negative concern for abuse. A score of -1 was assigned if the phrase was not found in the record, to prevent biasing the vector if a rule was merely inapplicable (i.e., the absence of a phrase associated with positive abuse concerns does not mean negative abuse concerns). Substantially more positive inflicted injury rules than negative rules were enumerated, since the absence of a finding is rarely recorded (e.g., if a rib fracture is present “rib fracture” will likely appear in the record, but if there is no rib fracture observed then the phrase generally does not appear).

### 2.3. Network design and training

MLP’s were implemented in Python 3 using the Keras environment [25]. Model weights were optimized using the ADAM optimizer and binary cross-entropy loss. Cross-validation was used across the study, to ensure that results were not biased by coincidental gathering of corpora that could be considered easily interpretable, and that each model was tested on unseen test data [26]. This process partitioned the dataset into 10 subsets, each with a distinct training and test-set of patients, with a random 10% of the training set used for internal validation during each training epoch. To prevent overfitting, the final model weights were chosen from the epoch with highest internal validation accuracy rather than automatically selecting the final epoch. After training, the model was evaluated on the held-out 10% test set. Initially, 4 architectures were chosen to test different numbers of hidden layers and dropout layers. For WE, accuracies were poor for all 4 architectures, so training was ceased. For BOW-TFIDF and RB, these results were used to create 4 additional architectures similar to the best-performing one from the original 4; allowing for further optimization. A total of 24 architectures were thus designed and trained on corpora: 8 each for BOW-TFIDF and RB, 4 each for WE-GLOVE and WE-MIMIC. The selected training parameters and architectures are shown in S1 Table and S2 Fig. Each model was trained and tested 10 independent times for each of 10 train-test splits, and classification results were compared to ground truth (the multi-disciplinary CAP Team assessment). The architecture within each encoding strategy with highest average test-set accuracy across the best model in each split was chosen.

For each chosen architecture, we calculated the average, standard deviation, and maximum accuracy and ROC-AUC for the best performing model weights from each split. The difference in the length of corpora between correct and incorrect classifications using BOW-TFIDF and RB models was tested for statistical significance. For the RB model, the difference in the number of invalid rules between correct and incorrect classifications was also tested. We also compared the results of the MLP using BOW and RB to those from a logistic regression using BOW and RB, in order to confirm the utility of a deep learning approach.

A saliency-frequency analysis for the best performing BOW-TFIDF model [27] was performed: for each test-set record, the numerical gradient across the network for each word in the corpus was calculated. Then the 50 words with the highest gradient were selected, and the frequency distribution of the 50 most frequently salient words for positive-abuse patients and negative-abuse patients in the test-set was constructed.

A leave-one-out sensitivity analysis for the best performing rules-based model was performed: each rule was invalidated in succession for all test-set records, and the change in accuracy for both the best and worst performing train-test splits was calculated.

The best performing BOW-TFIDF and RB models were applied to corpora created from MIMIC-III, using records of length similar to the mean length of abuse corpora. MIMIC is an adult ICU database, all records should have a negative classification for child abuse, and a positive model classification must be a false positive. The occurrence of each rule in MIMIC corpora was recorded, to identify reasons for false positives.

## 3. Results

**Fig 1:**
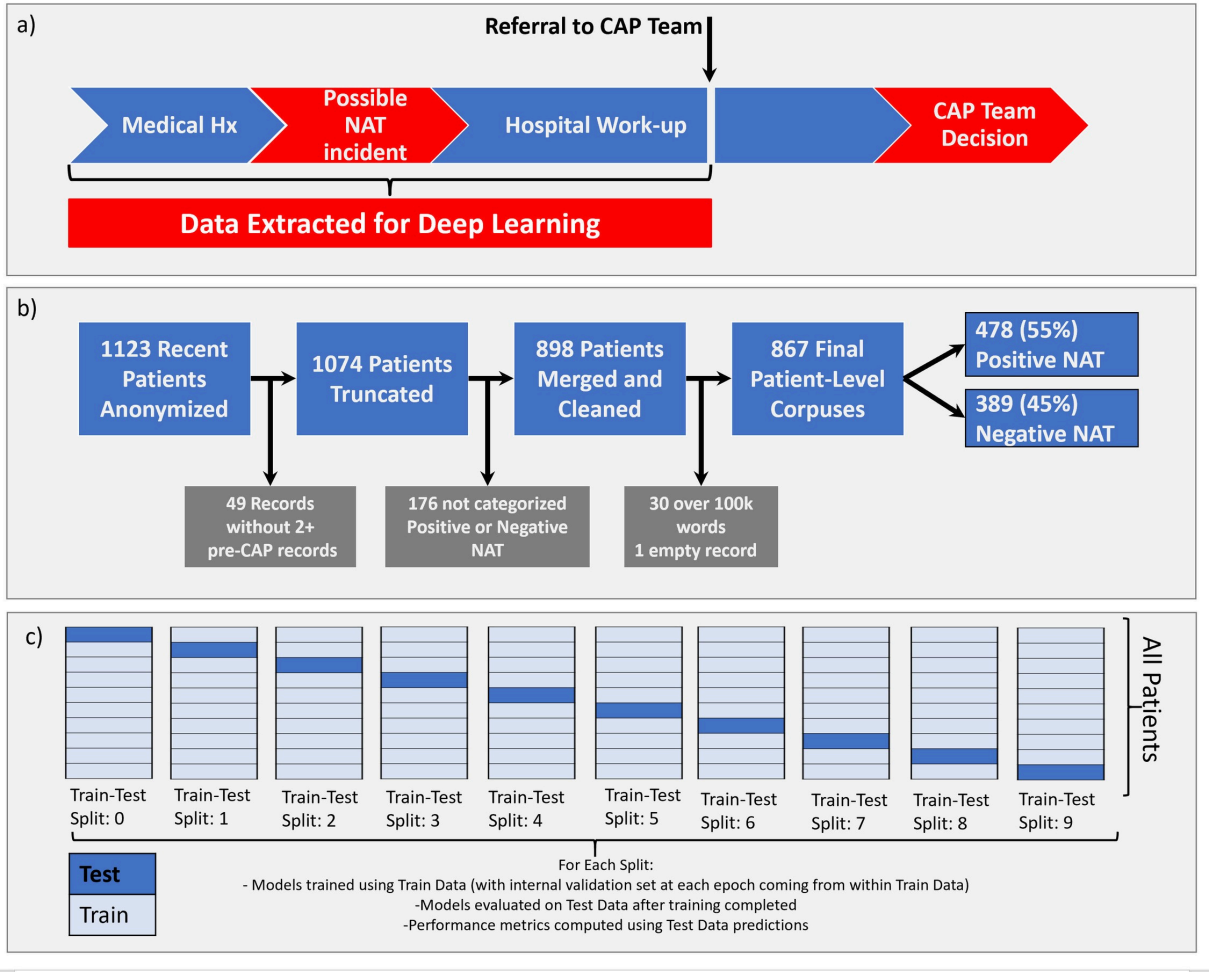
Schematic of patient record selection and processing. (a) The records were processed to extract only those notes written before the first note from a Child Abuse Pediatrics team MD or NP, hence allowing for prediction using only information available before the decision to refer a patient to the CAP team. (b) 1123 records for patients evaluated for suspected abuse between 1/1/2015 and 5/1/2019 were identified. Several were excluded for reasons listed in the figure, leaving 867 records for deep learning. (c) Schematic of cross-validation procedure used to create 10 distinct train-test splits.

From the initial 1123 patients, 49 were excluded with <2 notes after truncation. The remaining 1074 patients had 3–2145 notes (median = 21.5). The CAP team failed to classify 167 patients, leaving 898 for further processing. The median number of words was 6167.5 ranging from 0 to 994901. To accommodate memory requirements for MLP training, 30 patient corpora with > 100k words were excluded. One corrupted record was empty after data processing, and excluded, resulting in 867 patient records, 478 positive for physical abuse (55%) and 389 negative (45%) (Fig 1). This distribution of findings illustrates a methodical practice pattern in interpreting data to identify an unsafe caretaking environment despite the potential referral bias to the child abuse pediatrics service. Of these 867, the interval between first note and the truncation point (pre-CAP-Team-encounter) was 0–3381 (median 15) days. 8 of these patients were over the age of 5 years at CAP encounter, representing a more diverse age range than that used in other published study groups, such as those used to derive clinical prediction rules for abusive head trauma [3, 5].

**Fig 2:**
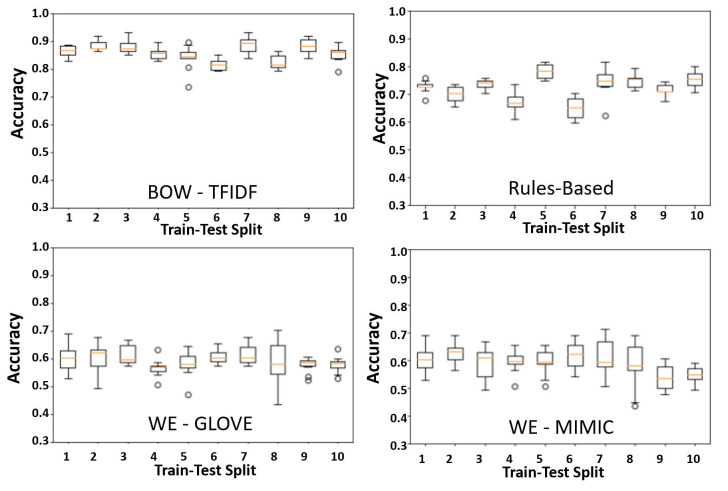
Cross- validation. Boxplots showing accuracy for n = 10 trials for each of 10 train-test splits, with our chosen model architecture in each strategy. The orange line shows the median, while the edges of the box show the 1st and 3rd quartile. The whiskers extend to 1.5 times the interquartile range, while points greater than 3rd quartile + 1.5*IQR or less than 1st quartile– 1.5*IQR are shown as discrete points.

**Fig 3:**
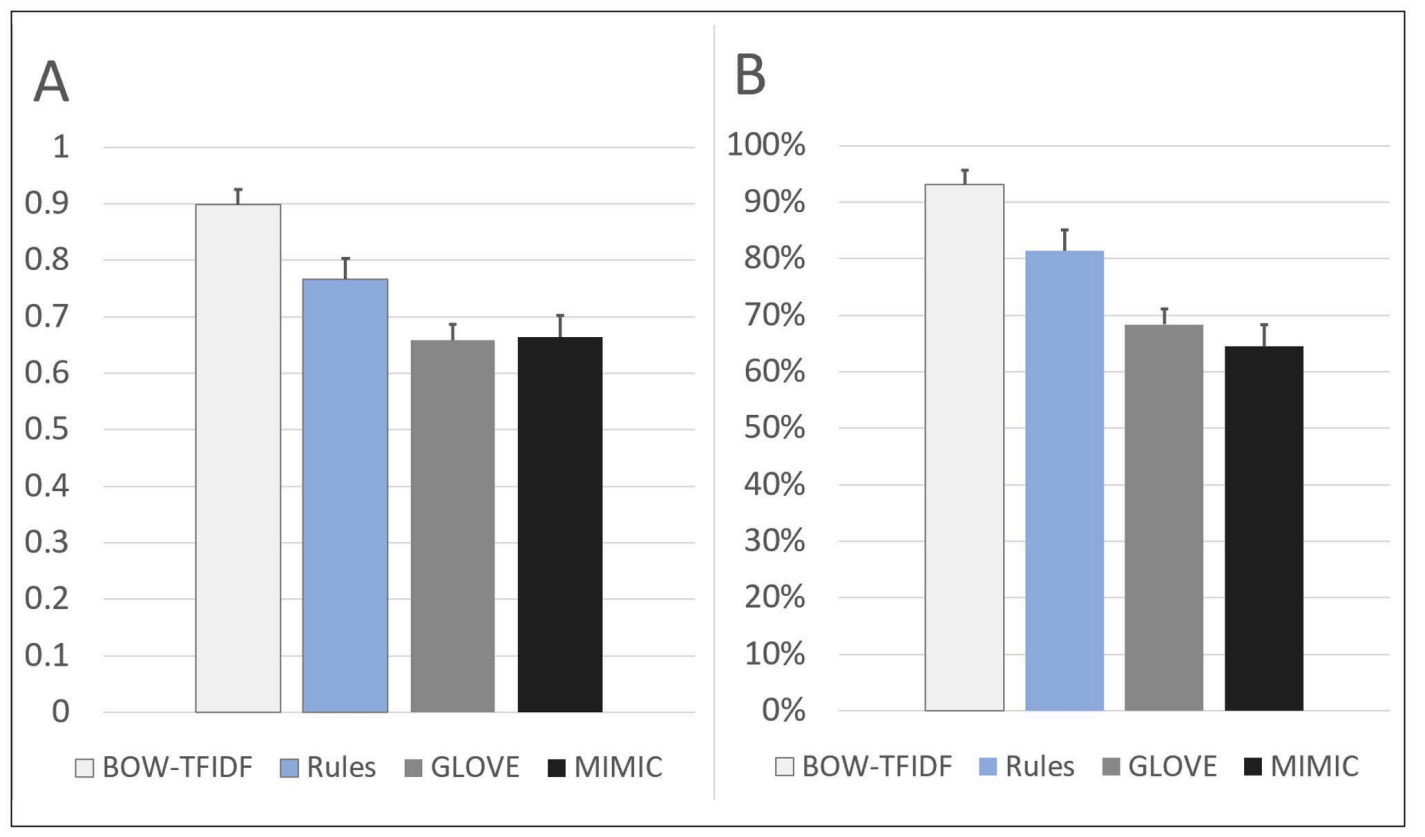
Performance of the best model in each of 10 train-test splits. A) the average accuracy of ten repetitions, B) the average area under the ROC curve (AUC) of ten repetitions.

**Fig 4:**
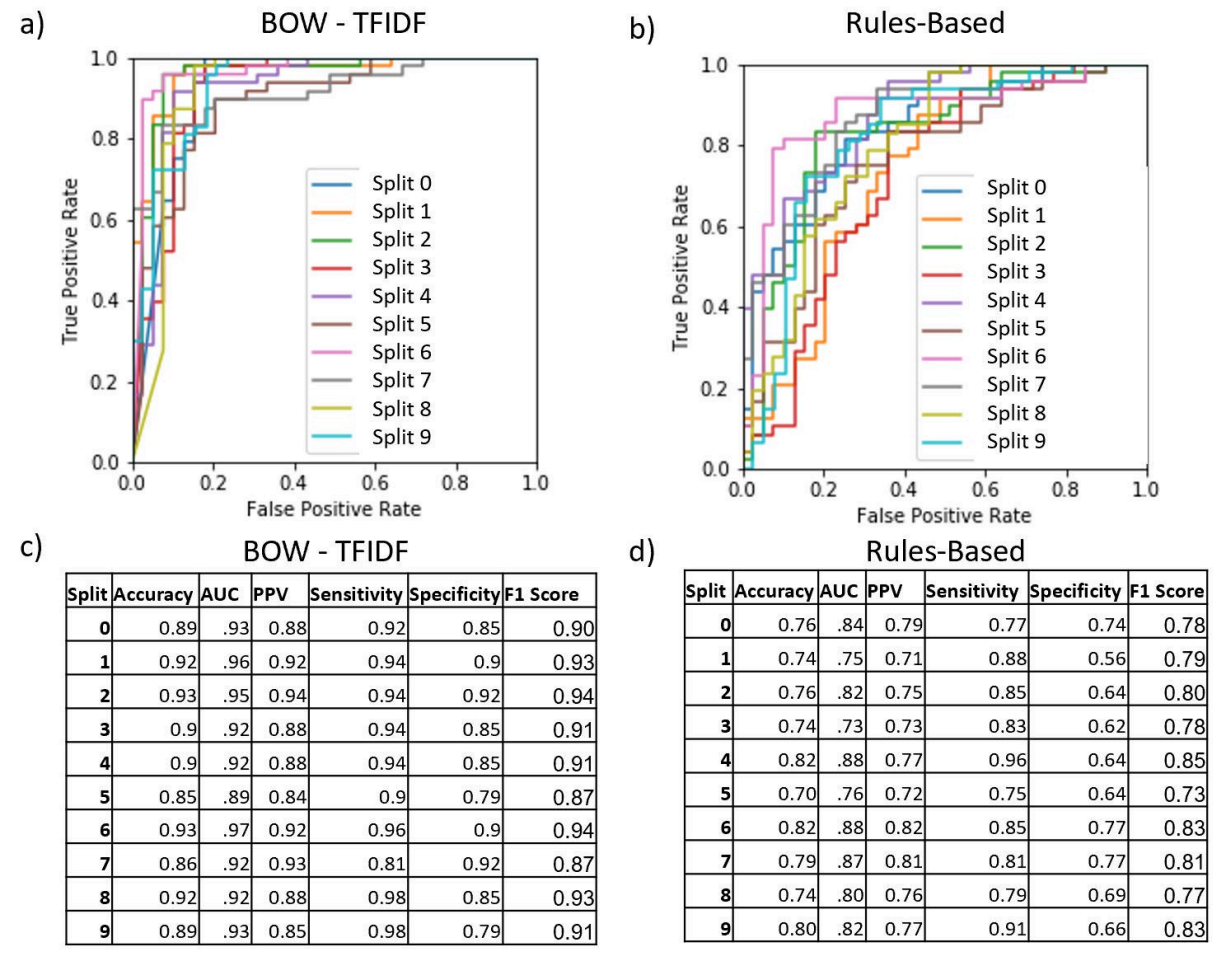
ROC curves, AUC, accuracy, PPV, sensitivity, specificity, and F1 score for the best performing model in each train-test split for BOW-TFIDF and rules-based approach. For each model category the receiver operator (ROC) curve, AUC, Accuracy, PPV, Sensitivity, Specificity, and F1 Score for the best model in each train-test split is shown. The ROC curve shows the sensitivity-specificity tradeoff for different classification thresholds, while the tables show the AUC for the ROC curve, as well as accuracy, PPV, sensitivity, specificity, and F1 score at the .5 threshold used in our classification algorithm. (a,c) BOW-TFIDF, (b,d) Rules-Based. The BOW models have highest AUC, with a characteristic ROC plot shape, and high sensitivity, PPV, specificity, and F1 Score.

The chosen model architectures and hyperparameters are summarized in S1 Table. The BOW-TFIDF and RB MLPs were run for 25 epochs (S2 Fig), while WE MLPs were run for 50 epochs, beyond which no improvements in accuracy were observed. The results of 10 repetitions of the chosen model architectures for each of 10 train-test splits are shown in Fig 2. The mean accuracy and AUC for the best performing model in each split (n = 10) are summarized in Fig 3. For the best model from each split by accuracy, the average accuracy (n = 10) was 89.9% (2.6% SD, max 93.1%) for BOW-TFIDF, 76.6% (3.7% SD, max 81.6%) for RB, 65.8% (SD 2.8%, max 70.1%) for WE-GLOVE, and 66.4% (SD 3.8%, max 71.2%) for WE-MIMIC. The average ROC-AUC was 93.1% (2.2% SD) for BOW-TFIDF, 81.4% (5.2% SD) for RB, 68.3% (SD 3.5%) for WE-GLOVE, and 64.5% (SD 7.5%) for WE-MIMIC. The WE results are limited by the lack of representative embedding–only 51% and 46% of words in the corpora were contained in the WE-GLOVE and WE-MIMIC embeddings, respectively. For BOW-TFIDF and RB, the ROC plots and associated AUC, along with Sensitivity, Specificity, Accuracy, and Positive Predictive Value (PPV) for the best performing model from each train-test split are shown in Fig 4.

The logistic regression models on average across the 10 different train-test splits had 1% lower accuracy, and 5% lower AUC for BOW than the MLP models (S2 Table). These differences were even larger for RB, with 4% lower accuracy and 10% lower AUC on average for logistic regression as compared to MLP (S2 Table). While the differences in accuracy between logistic regression and deep learning may seem minor, the AUC differences are quite large. For our application, where sensitivity and specificity are crucial, the large AUC improvement shown with deep learning demonstrates its greater utility as compared to logistic regression.

**Fig 5:**
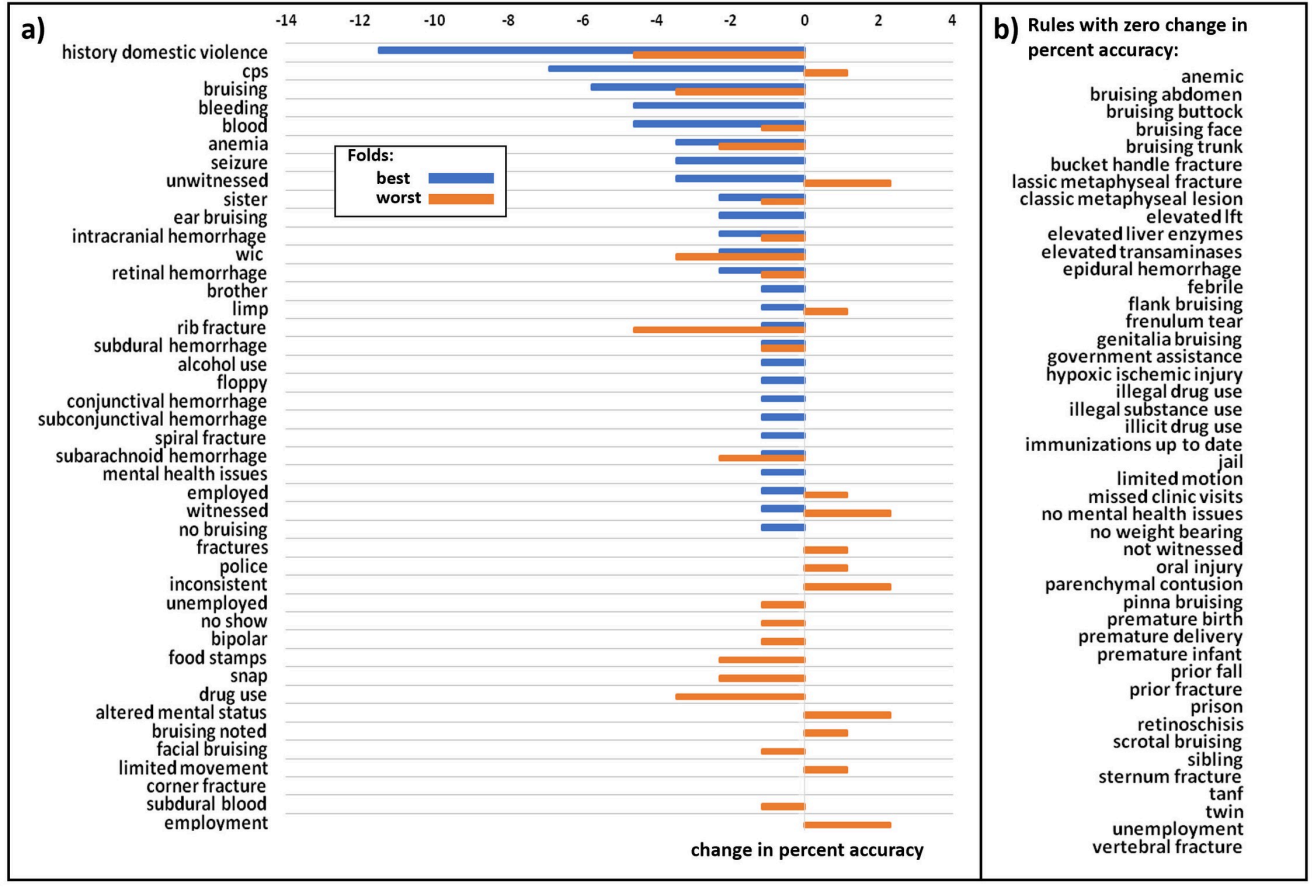
Leave-one-out sensitivity analysis of rules used in rules-based approach. (a) The change in percentage accuracy that occurs when each rule is in invalidated (set to -1 for each record) for the best performing model from the best train-test split by maximum accuracy, and the best performing model from the worst train-test split by maximum accuracy. For the best split, the invalidation of each rule has no change or lowers the accuracy, with the phrase “history domestic violence” having the greatest impact and reducing accuracy by 0.11 from 0.82 to 0.7. For the worst split, the invalidation of each rule can have no change, or can raise or lower the accuracy. The phrases “history domestic violence” and “rib fracture” have the largest negative impact and reduce accuracy by 0.05 from 0.7 to 0.65, while the phrases “Inconsistent”, “unwitnessed”, “altered mental status”, “employment” and “witnessed” have the largest positive impact and increase accuracy by 0.02 to 0.72. (b) Alphabetical list of rules which do not change accuracy during leave-one-out sensitivity analysis.

The frequency distribution of the top 50 most salient words from the positive-abuse and negative-abuse test cases for the best performing BOW-TFIDF model (by accuracy) is shown in S3 Fig. Fig 5 shows the results of the leave-one-out sensitivity analysis for the best-performing RB model, with the invalidation of the phrase “history domestic violence” corresponding to the greatest decrease in accuracy. A two-tailed t-test for difference of means on the best performing model in each train-test split showed no significant difference in length of records for either the BOW-TFIDF models (p = .095) or RB models (p = .71) (S4 Fig). No significant difference appeared in the number of invalid rules (rules whose phrase was not found in the record) between the patients correctly and incorrectly classified by the RB models (p = .97) in a two-tailed t-test for difference of means (S5 Fig). However, the distribution of predicted probability of abuse between incorrectly and correctly classified patients differed significantly for both the BOW-TFIDF and RB techniques (p = 7.17e-7 for BOW-TFIDF classifications and p = .0002 for RB classifications) by a chi-squared contingency test. For correctly classified patients the predicted probabilities fall in a bimodal distribution, whereas probabilities for incorrectly classified patients distribute uniformly (S6A and S6B Fig).

When run on the MIMIC database of adult ICU patients with records between 5000 and 7000 words in length (which must all be negative for the physical abuse of a child), the BOW-TFIDF model classifies 3740 out of 4611 (81.1%) as positive. The RB model classifies 3210 out of 4611 (69.6%) as positive. Analyzing the applicability of the rules to the MIMIC database shows that the majority of the rules are present in greatly different proportions of the MIMIC records than the maltreatment records, and that the phrases have different meanings in the context of adult ICU patients versus pediatric abuse patients (S7 Fig).

## 4. Discussion

The BOW-TFIDF and RB approaches resulted in high classification accuracy and high ROC-AUC (Figs 3 and 4). The accuracies and AUCs are similar across train-test splits (Fig 2), demonstrating that selected model architectures are consistently suitable—even with different training and test set partitions—and the training procedure is robust and reproducible despite the stochasticity inherent in deep learning. The WE based models had substantially weaker results than BOW-TFIDF and RB, likely due to the small percentage of words embedded (Figs 2 and 3).

For an additional assessment of misclassifications, we selected 25 cases randomly out of those misclassified by the BOW model for expert physician assessment. Here, we noted 4 reasons for misclassification. Among the 6 false negative cases, all were in patients under 7 months with minimal medical records requiring further substantiation. Among the 19 false positive cases, all were in three groups: (1) The abuse finding was based on neglect, (2) There was no documented mechanism of injury in the notes prior to CAP team involvement, making the dataset presented to the algorithm incomplete, or (3) There were multiple injuries and evidence collected did not support all the injuries; therefore, more information was requested after CAP Team involvement which was not presented to the algorithm. These reasons all reflect complicated circumstances beyond concrete physical abuse, and in our assessment would also be difficult for clinicians to make an accurate determination. For many of these cases, the network predicted an intermediate probability reflective of relative uncertainty in the prediction (S6C Fig).

Moreover, our BOW-TFIDF models have comparable or better accuracy to previously reported models [17] despite our decision to eschew terminology normalization or ensembling with a feature selection model. This ensures that our methods are easily deployable without time-intensive, often manual pre-processing steps. While the study with records from the Netherlands employed conventional machine learning methods (random forest, support vector machines etc.) and we employed deep learning techniques (MLP), both studies found BOW-TFIDF to be a successful encoding technique. This utility of BOW-TFIDF across records from both the Netherlands [17] and the United States (present study) utilizing different natural languages (Dutch vs. English) indicates the robust nature of the encoding technique. This previous study reported a best AUC of .914 with an accuracy of .822 for a single ensembled model that used both structured and unstructured features. In contrast, our single best performing BOW-TFIDF model (unstructured features only) achieved both higher AUC (.95) and accuracy (.93). Of particular note, our model maintains high sensitivity and specificity (0.94 and 0.92), which corresponds to an 8% false positive rate. This is a substantial improvement over prior work with a ~20% FPR [4].

### 4.1. Strengths and limitations

The saliency-frequency map (S3 Fig) of the BOW-TFIDF network demonstrates several interesting features. The word “no” had high saliency regardless of classification, since the inclusion or exclusion of the word “no” directly reverses meaning. Other words, like “trauma,” “head,” and “negative” were highly salient in both abuse-positive and abuse-negative records for the same reason–their presence is important to the network’s classification. In contrast, some terms like “CPS” and “weight” or “kg” were highly salient only in positive maltreatment records. Since saliency measures how changing a given word would change the classification, reliance on clearly relevant words shows promise for this model.

The RB approach is also encouraging–while the accuracy and ROC-AUC are lower than BOW-TFIDF, it has the advantage of incorporating clinician input in determining the rules. This could refine practice by tailoring rules for community-network hospitals, and taking into account the expertise of CAP-trained physicians. However, formulating and validating these rules takes manual time and effort in contrast to BOW-TFIDF, which provides an end-to-end automated pipeline with comparable or better results.

A significant drawback of the RB approach is its potential to emphasize socioeconomic biases in the diagnosis of child abuse, through its inclusion of rules reflecting clinicians’ perceptions of a child’s social circumstances. However, our sensitivity analysis (Fig 5) showed that “history domestic violence” had the most significant impact on classification. Importantly, interpersonal violence has been well described as a multi-directional phenomenon within a household, putting not only partners but minor household members at risk for harm [28–30]. Therefore, it captures an important adverse childhood experience associated with abuse, without relying on potentially biased racial, ethnic, or socioeconomic linkages. Invalidation of this rule reduced accuracy by 0.11 from 0.82 to 0.7 for the best performing model in the best train-test split and reducing accuracy by 0.05 from 0.7 to 0.65 for the best performing model in the worst train-test split.

BOW-TFIDF and RB techniques are highly context-specific to exclusively patients for whom there is already a concern for abuse. Application of the best-performing BOW-TFIDF model to the MIMIC Adult ICU patient records yielded an 81.1% false-positive rate. TFIDF calculations performed on the records of suspected abuse patients during the training of the model did not appear to apply to the records of Adult ICU patients, where similar words could be used in different contexts. Using the best-performing RB model on the MIMIC Adult ICU patient records led to an 69.6% false-positive rate. We suspect this occurred because rules formulated in the child maltreatment context were confusing in the adult ICU context. For example, a phrase like “vertebral fracture” frequently occurred in both sets; however, that clinical finding is more likely to raise concern for abuse in pediatric settings. Moreover, some phrases worrisome for child maltreatment such as “illicit drug use” and “limited motion,” occur in a far greater percentage of MIMIC records than pediatric records (S7 Fig), further illustrating that the MIMIC records are not well suited for evaluation using a model explicitly trained on child abuse records and intended to be used as an aid for identifying pediatric patients for referral to the CAP team.

Only ~50% of the words in our corpora were represented in the WE-GLOVE and WE-MIMIC embeddings. This demonstrates the importance of utilizing embeddings trained on a corpus with a vocabulary representative of the corpora which are being classified (Wikipedia and Adult ICU notes vs. pediatric injury notes, in the present study). Word Embeddings are generally regarded as a top-performing NLP encoding technique; therefore, the lack of a suitable embedding is a significant limitation to the utilization of the technique in this context.

Another limitation of this study is the lack of cross-institutional validation. Due to the context-specific nature of the encoding techniques, it will be important to test and refine this pipeline in the context of EMRs from other institutions, which likely contain syntax specific to their clinical teams and specific patient populations. However, in the context of this work, we demonstrate that a natural language processing and deep learning pipeline can be effectively utilized to develop an abuse detection system for patients seen at both the main clinical location and satellite referral sites.

In the future, the development of representative embeddings will be required to harness the power of word embeddings for child physical abuse classification. This may be done with a larger corpus of pediatric electronic medical records, separate from the records concerning child abuse. Incorporating direct reports from laboratory and imaging data into the deep learning approach offers another opportunity to use the objective findings to improve the accuracy and transferability of the network. Innovative approaches to combine BOW-TFIDF inputs with word embeddings, or to use phrases rather than individual words as inputs hence preserving more syntactical meaning, may also yield improvements to algorithmic performance. Finally, alternative deep learning models can also be used to increase classification accuracy—these include alternative architectures such as Long Short-Term Memory (LSTM) and Recurrent Neural Networks (RNN) that have shown promise in text classification applications [31, 32]. Finally, efforts to implement, utilize and refine these deep learning approaches in a clinical setting will be crucial to improving performance and applicability.

## 5. Conclusions

This study was aimed at creating a screening algorithm for pediatric trauma cases in order to identify those children whose safety could be improved by a more extensive consideration of child maltreatment as a cause of their presenting injuries. In order to re-create a practice environment in which inclusion of child physical abuse is not commonly included in the differential diagnosis, deep learning approaches to classify inflicted injury to children were developed using only the portions of the electronic medical record before referral to a CAP team. These analyses achieved average accuracy of 0.90 and average ROC-AUC of 0.93 (for the best performing NLP processing technique, BOW-TFIDF), a combination that represents a significant advance over those obtained by non-deep learning approaches and other published studies of clinical prediction rules focused on an age group or specific diagnosis within the category of physical abuse [3–5, 17, 33–35]. The application of deep learning approaches to natural language processing of the free-text provider notes in electronic medical records could be used as a computer-aided diagnosis system, to identify patients with a high likelihood of abuse for a referral to trained CAP physicians, and may offer the potential to identify children at risk for abuse proactively and in real time. Such an approach could help providers act more readily to identify occult injuries in the index patient and also could assist in recruiting community partners for early involvement both in home assessments and safety evaluations for other children sharing the care environment where the injury occurred.

## Supporting information

S1 Fig CAP team referral process. Cases are referred to the CAP team from numerous sources including hospital clinical departments, social workers, Child Protective Services, and Community Pediatricians at the hospital’s satellite referral sites. The models described in this paper successfully process free-text notes from all of these types of patient encounters.
(DOCX)

S2 Fig Training and validation loss curves. (a) Bag of Words, 10 models from best train-test split as chosen by maximum accuracy, (b) Bag of Words, 10 models from worst train-test split as chosen by maximum accuracy, (c) Rules-based, 10 models from best train-test split as chosen by maximum accuracy, (d) Rules-based, 10 models from worst train-test split as chosen by maximum accuracy. Over the first 5 epochs, the training loss decreases, while validation loss stabilizes and then increases as the model moves towards being overfit. Training was performed over a total of 25 epochs were chosen to ensure we trained past the inflection point between training and validation loss. However, to prevent overfitting, the epoch with the highest validation accuracy was then selected for the final model weights.
(DOCX)

S3 Fig Saliency for BOW-TFIDF. The frequency distribution of the 50 most salient words across the abuse-positive and abuse-negative test cases is shown for the best performing BOW-TFIDF model. Saliency refers to the words which, if changed, would have the greatest impact on classification. The top 50 most salient words for each test case patient were calculated, and then a frequency distribution of these words was created, with the top 50 most frequent words shown in the (a) abuse-positive cases and (b) abuse-negative cases.
(DOCX)

S4 Fig Length distributions. Distribution of cleaned patient record length vs the best performing model in each train-test split’s predicted probability of NAT for (a) Bag of Words, Correct predictions, (b) Bag of Words, Incorrect predictions, (c) Rules-Based, Correct predictions, and (d) Rules-Based, Incorrect predictions. The difference between lengths of correctly classified and incorrectly classified records is not significant by a two-tailed t-test, for either approach (p = .095 for Bag of Words, p = .71 for Rules-based). For correct predictions, probabilities greater than or equal to .5 correspond to true positives, and probabilities below .5 correspond to true negatives. For incorrect predictions, probabilities greater than or equal to .5 correspond to false positives, and probabilities below .5 correspond to false negatives.
(DOCX)

S5 Fig Number of invalid rules distribution. Distribution of number of rules (of 88) invalid (phrase not found in the record) vs. the best performing Rules-based model in each train-test split’s predicted probability of NAT for (a) Correctly classified patients, and (b) Incorrectly classified patients. The difference in number of invalid rules between the Correct and Incorrect predictions is statistically insignificant by a two-tailed t-test (p = .97). For correct predictions, probabilities greater than or equal to .5 correspond to true positives, and probabilities below .5 correspond to true negatives. For incorrect predictions, probabilities greater than or equal to .5 correspond to false positives, and probabilities below .5 correspond to false negatives.
(DOCX)

S6 Fig Distributions of predicted probabilities of NAT from the best performing model in each train-test split for rules-based and bag of words. (a) BOW-TFIDF classifications and (b) Rules-based classifications–Qualitatively, the models predict probabilities near 0 and 1 for correct predictions and have a greater range for incorrect predictions. The differences in distribution are statistically significant by a chi-squared contingency test (p = 7.17e-7 for BOW-TFIDF classifications and p = .0002 for Rules-based classifications).
(DOCX)

S7 Fig Ratio of proportion of MIMIC records containing rule to proportion of NAT records containing rule. Proportions greater than 1 refer to phrases that were much more prevalent in the MIMIC records than in the NAT records. For example “illicit drug use” and”blood” occurred in 18.3 and 1.2 times more MIMIC records respectively. In the context of the MIMIC records (taken from adult ICU patients) these phrases are not relevant to NAT, but in the training of our models on NAT-specific records, these words were designated as indicative of positive NAT. This disparity presents a potential reason why the rules-based model has a high false positive rate when tested on the MIMIC database, and highlights the context dependence of the models presented in this paper.
(DOCX)

S1 Table Architecture and hyperparameters for the chosen model in each strategy. (DOCX)

S2 Table Comparison of logistic regression vs. MLP performance with BOW and RB encodings. (DOCX)
