# Supplementary material for: A natural language processing and deep learning approach to identify child abuse from pediatric electronic medical records
Source: PLoS One. 2021 Feb 26;16(2):e0247404. doi: 10.1371/journal.pone.0247404 (PMC7909689; doi:10.1371/journal.pone.0247404)
Supplement: S1 Fig — Cases are referred to the CAP team from numerous sources including hospital clinical departments, social workers, Child Protective Services, and Community Pediatricians at the hospital’s satellite referral sites. The models described in this paper successfully process free-text notes from all of these types of patient encounters. (DOCX) [file pone.0247404.s001.docx]

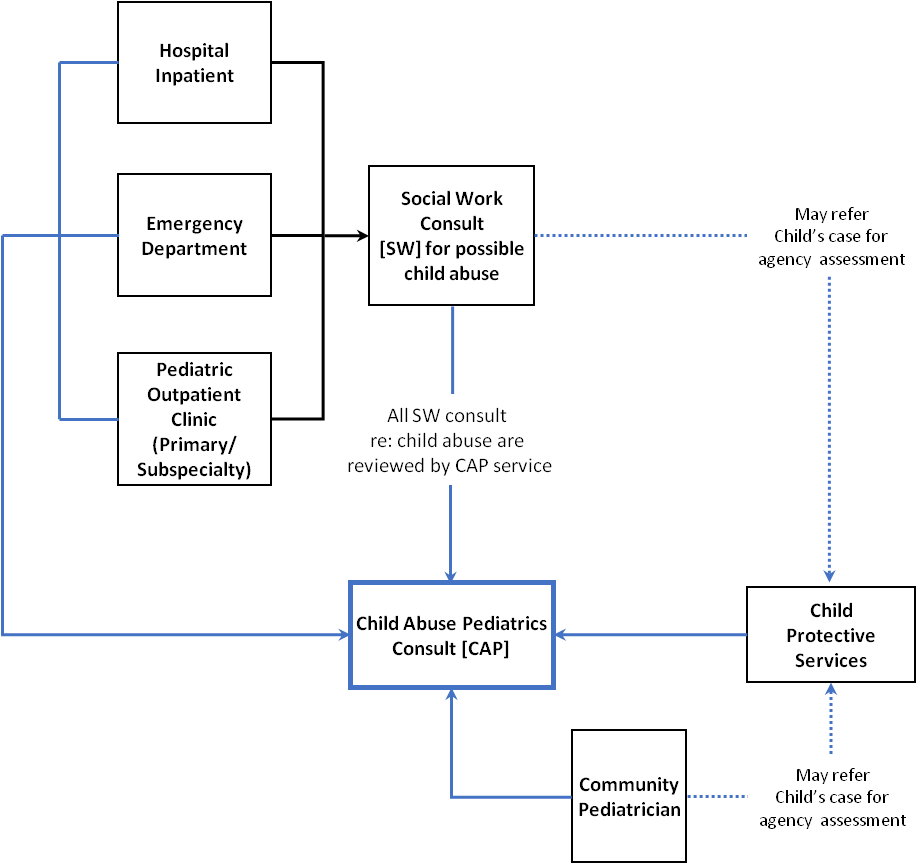


**S1 Fig. CAP Team Referral Process**– Cases are referred to the CAP team from numerous sources including hospital clinical departments, social workers, Child Protective Services, and Community Pediatricians at the hospital’s satellite referral sites. The models described in this paper successfully process free-text notes from all of these types of patient encounters.
