# Supplementary material for: A natural language processing and deep learning approach to identify child abuse from pediatric electronic medical records
Source: PLoS One. 2021 Feb 26;16(2):e0247404. doi: 10.1371/journal.pone.0247404 (PMC7909689; doi:10.1371/journal.pone.0247404)
Supplement: S2 Fig — (a) Bag of Words, 10 models from best train-test split as chosen by maximum accuracy, (b) Bag of Words, 10 models from worst train-test split as chosen by maximum accuracy, (c) Rules-based, 10 models from best train-test split as chosen by maximum accuracy, (d) Rules-based, 10 models from worst train-test split as chosen by maximum accuracy. Over the first 5 epochs, the training loss decreases, while validation loss stabilizes and then increases as the model moves towards being overfit. Training was performed over a total of 25 epochs were chosen to ensure we trained past the inflection point between training and validation loss. However, to prevent overfitting, the epoch with the highest validation accuracy was then selected for the final model weights. (DOCX) [file pone.0247404.s002.docx]

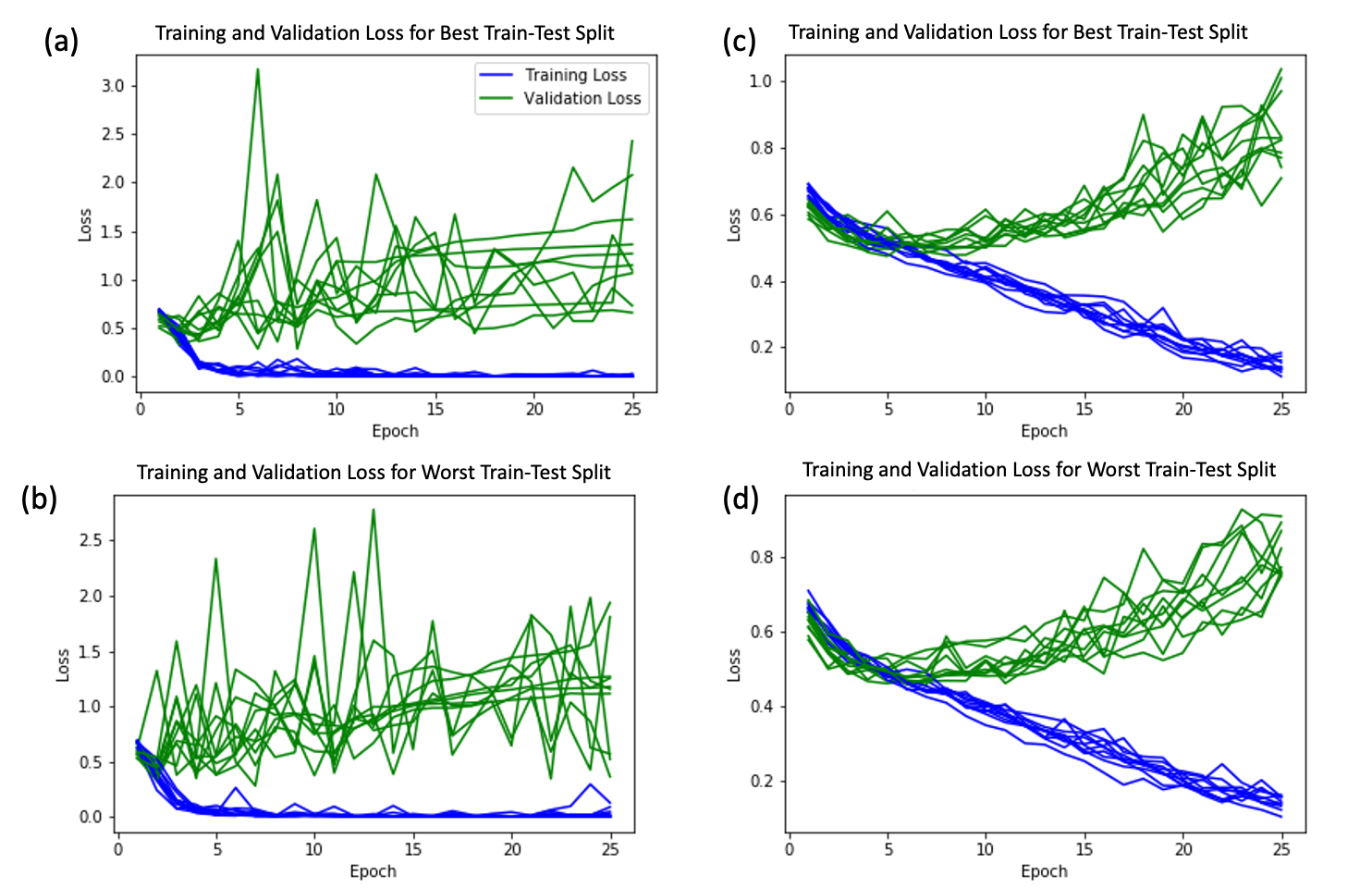


**S2 Fig. Training and Validation Loss Curves** – (a) Bag of Words, 10 models from best train-test split as chosen by maximum accuracy, (b) Bag of Words, 10 models from worst train-test split as chosen by maximum accuracy, (c) Rules-based, 10 models from best train-test split as chosen by maximum accuracy, (d) Rules-based, 10 models from worst train-test split as chosen by maximum accuracy. Over 25 epochs, the training loss decreases, while validation loss stabilizes and then increases as the model moves towards being overfit. 25 epochs was chosen to ensure we trained past the inflection point between training and validation loss. However, to prevent overfitting, the epoch with the highest validation accuracy was then selected as the final model weights
