# Supplementary material for: A natural language processing and deep learning approach to identify child abuse from pediatric electronic medical records
Source: PLoS One. 2021 Feb 26;16(2):e0247404. doi: 10.1371/journal.pone.0247404 (PMC7909689; doi:10.1371/journal.pone.0247404)
Supplement: S3 Fig — The frequency distribution of the 50 most salient words across the abuse-positive and abuse-negative test cases is shown for the best performing BOW-TFIDF model. Saliency refers to the words which, if changed, would have the greatest impact on classification. The top 50 most salient words for each test case patient were calculated, and then a frequency distribution of these words was created, with the top 50 most frequent words shown in the (a) abuse-positive cases and (b) abuse-negative cases. (DOCX) [file pone.0247404.s003.docx]

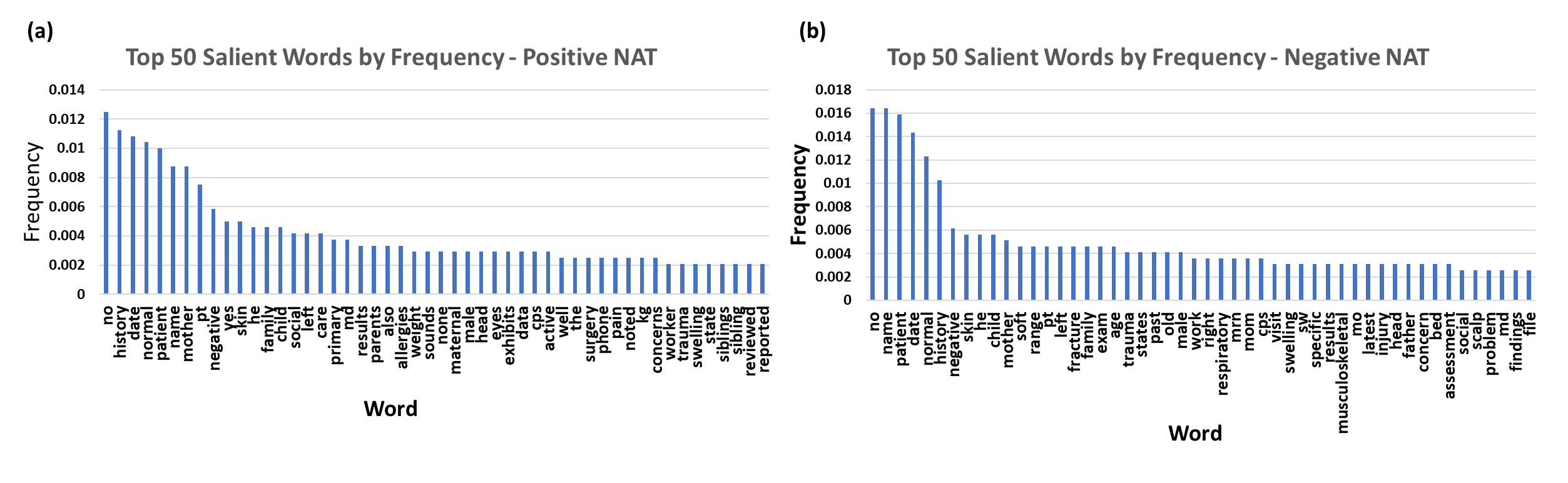


**S3 Fig. Saliency for BOW-TFIDF -** The frequency distribution of the 50 most salient words across the positive-NAT and negative-NAT test cases is shown for the best performing BOW-TFIDF model. Saliency refers to the words which, if changed, would have the greatest impact on classification. The top 50 most salient words for each test case patient were calculated, and then a frequency distribution of these words was created, with the top 50 most frequent words shown in the (a) Positive NAT cases and (b) Negative NAT cases.
