# Supplementary material for: A natural language processing and deep learning approach to identify child abuse from pediatric electronic medical records
Source: PLoS One. 2021 Feb 26;16(2):e0247404. doi: 10.1371/journal.pone.0247404 (PMC7909689; doi:10.1371/journal.pone.0247404)
Supplement: S4 Fig — Distribution of cleaned patient record length vs the best performing model in each train-test split’s predicted probability of NAT for (a) Bag of Words, Correct predictions, (b) Bag of Words, Incorrect predictions, (c) Rules-Based, Correct predictions, and (d) Rules-Based, Incorrect predictions. The difference between lengths of correctly classified and incorrectly classified records is not significant by a two-tailed t-test, for either approach (p = .095 for Bag of Words, p = .71 for Rules-based). For correct predictions, probabilities greater than or equal to .5 correspond to true positives, and probabilities below .5 correspond to true negatives. For incorrect predictions, probabilities greater than or equal to .5 correspond to false positives, and probabilities below .5 correspond to false negatives. (DOCX) [file pone.0247404.s004.docx]

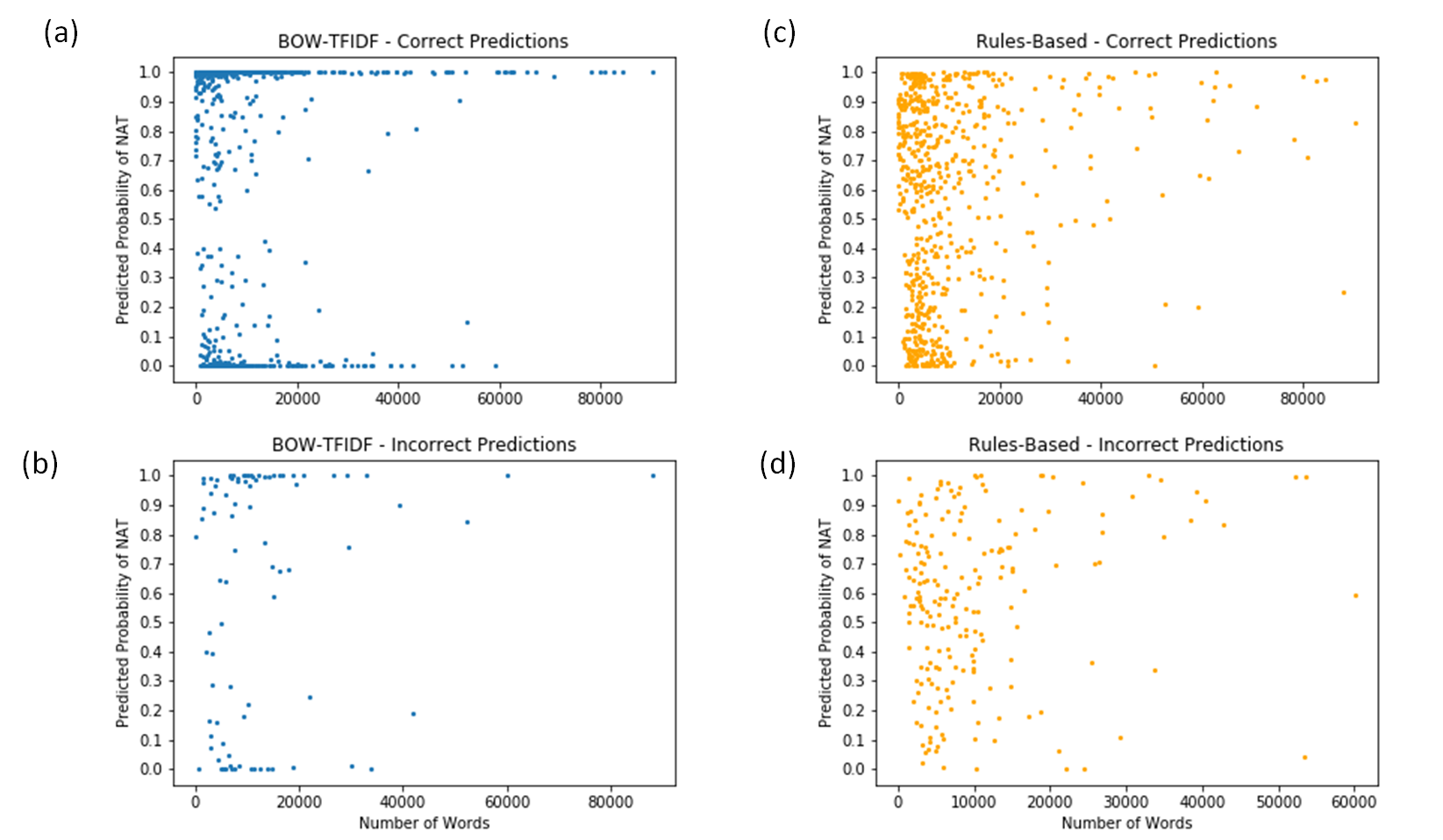


**S4 Fig. Length distributions –** Distribution of cleaned patient record length vs the best performing model in each train-test split’s predicted probability of NAT for (a) Bag of Words, Correct predictions, (b) Bag of Words, Incorrect predictions, (c) Rules-Based, Correct predictions, and (d) Rules-Based, Incorrect predictions. The difference between lengths of correctly classified and incorrectly classified records is not significant by a two-tailed t-test, for either approach (p=.095 for Bag of Words, p=.71 for Rules-based). For correct predictions, probabilities greater than or equal to .5 correspond to true positives, and probabilities below .5 correspond to true negatives. For incorrect predictions, probabilities greater than or equal to .5 correspond to false positives, and probabilities below .5 correspond to false negatives.
