# Supplementary material for: A natural language processing and deep learning approach to identify child abuse from pediatric electronic medical records
Source: PLoS One. 2021 Feb 26;16(2):e0247404. doi: 10.1371/journal.pone.0247404 (PMC7909689; doi:10.1371/journal.pone.0247404)
Supplement: S5 Fig — Distribution of number of rules (of 88) invalid (phrase not found in the record) vs. the best performing Rules-based model in each train-test split’s predicted probability of NAT for (a) Correctly classified patients, and (b) Incorrectly classified patients. The difference in number of invalid rules between the Correct and Incorrect predictions is statistically insignificant by a two-tailed t-test (p = .97). For correct predictions, probabilities greater than or equal to .5 correspond to true positives, and probabilities below .5 correspond to true negatives. For incorrect predictions, probabilities greater than or equal to .5 correspond to false positives, and probabilities below .5 correspond to false negatives. (DOCX) [file pone.0247404.s005.docx]

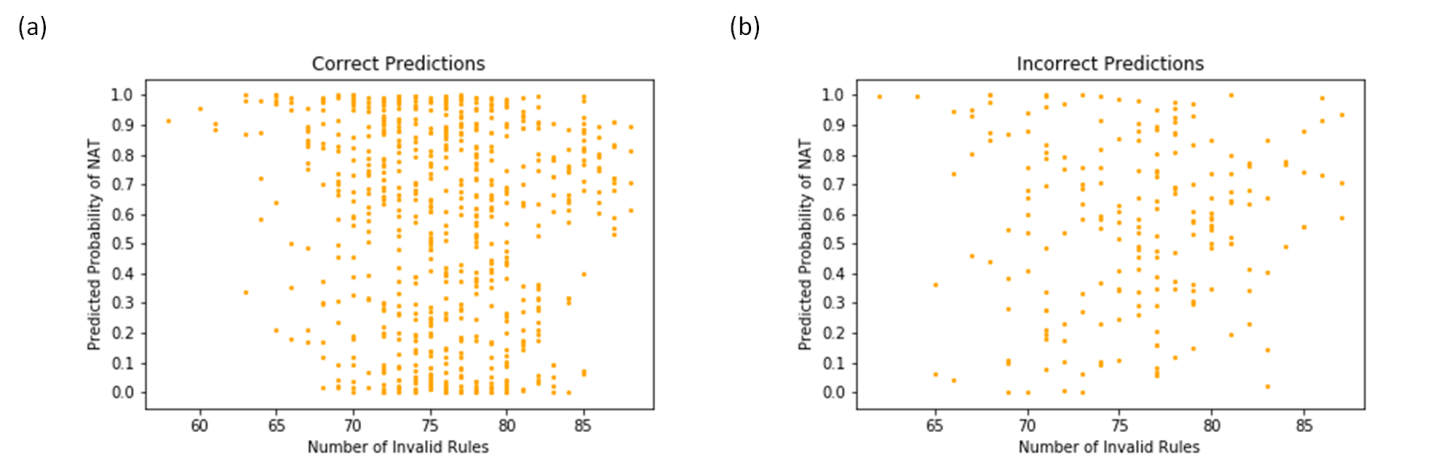


**S5 Fig. Number of Invalid Rules Distribution–** Distribution of number of rules (of 88) invalid (phrase not found in the record) vs. the best performing Rules-based model in each train-test split’s predicted probability of NAT for (a) Correctly classified patients, and (b) Incorrectly classified patients. The difference in number of invalid rules between the Correct and Incorrect predictions is statistically insignificant by a two-tailed t-test (p=.97). For correct predictions, probabilities greater than or equal to .5 correspond to true positives, and probabilities below .5 correspond to true negatives. For incorrect predictions, probabilities greater than or equal to .5 correspond to false positives, and probabilities below .5 correspond to false negatives.
