# Supplementary material for: A natural language processing and deep learning approach to identify child abuse from pediatric electronic medical records
Source: PLoS One. 2021 Feb 26;16(2):e0247404. doi: 10.1371/journal.pone.0247404 (PMC7909689; doi:10.1371/journal.pone.0247404)
Supplement: S6 Fig — (a) BOW-TFIDF classifications and (b) Rules-based classifications–Qualitatively, the models predict probabilities near 0 and 1 for correct predictions and have a greater range for incorrect predictions. The differences in distribution are statistically significant by a chi-squared contingency test (p = 7.17e-7 for BOW-TFIDF classifications and p = .0002 for Rules-based classifications). (DOCX) [file pone.0247404.s006.docx]

**S6 Fig. Distributions of Predicted Probabilities of NAT from the best performing model in each train-test split for Rules-Based and Bag of Words**, and for a random set of 25 misclassified patients (a) BOW-TFIDF classifications and (b) Rules-based classifications – Qualitatively, the models predict probabilities near 0 and 1 for correct predictions and has a greater range for incorrect predictions. The differences in distribution are statistically significant by a chi-squared contingency test (p=7.17e-7 for BOW-TFIDF classifications and p=.0002 for Rules-based classifications). (c) Predicted probabilities for 25 cases predicted incorrectly by the BOW Model – several of the misclassifications had intermediate predicted probabilities, reflecting uncertainty in misclassification.
