# Supplementary material for: A natural language processing and deep learning approach to identify child abuse from pediatric electronic medical records
Source: PLoS One. 2021 Feb 26;16(2):e0247404. doi: 10.1371/journal.pone.0247404 (PMC7909689; doi:10.1371/journal.pone.0247404)
Supplement: S7 Fig — Proportions greater than 1 refer to phrases that were much more prevalent in the MIMIC records than in the NAT records. For example “illicit drug use” and”blood” occurred in 18.3 and 1.2 times more MIMIC records respectively. In the context of the MIMIC records (taken from adult ICU patients) these phrases are not relevant to NAT, but in the training of our models on NAT-specific records, these words were designated as indicative of positive NAT. This disparity presents a potential reason why the rules-based model has a high false positive rate when tested on the MIMIC database, and highlights the context dependence of the models presented in this paper. (DOCX) [file pone.0247404.s007.docx]

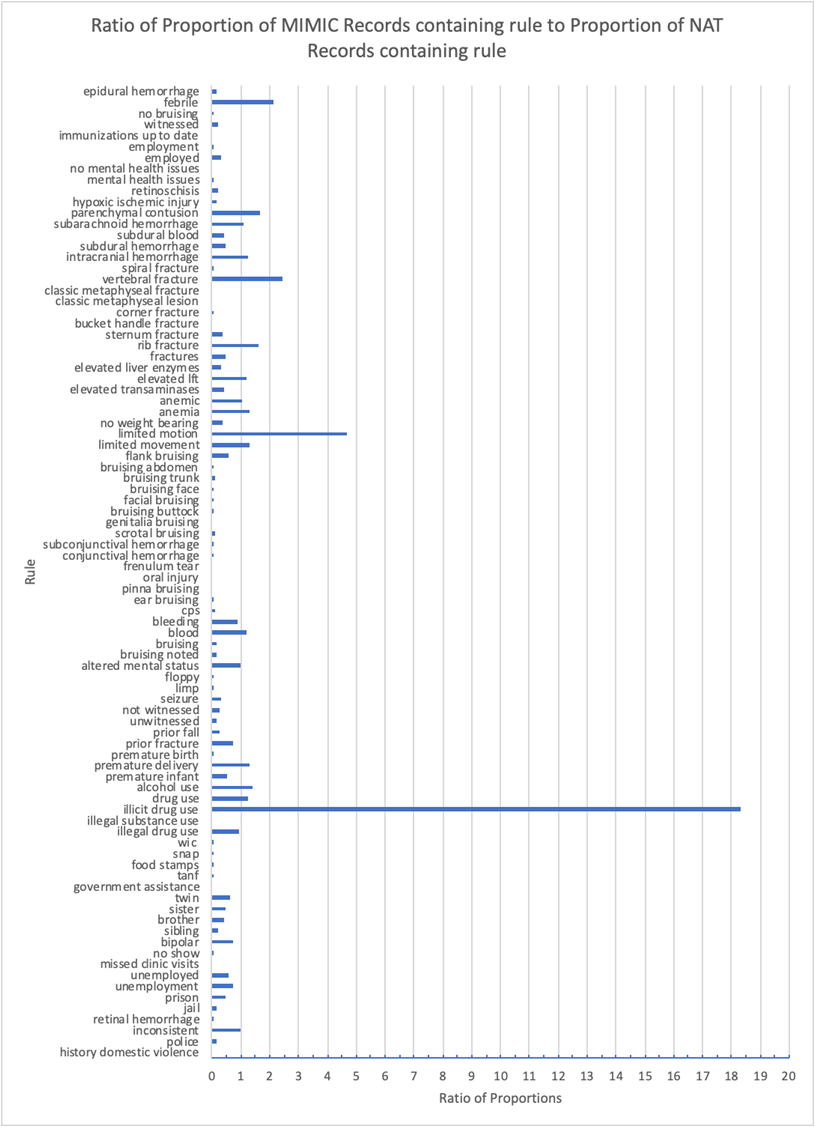


**S7 Fig. Ratio of Proportion of MIMIC Records containing rule to proportion of NAT Records containing rule –** Proportions greater than 1 refer to phrases that were much more prevalent in the MIMIC records than in the NAT records. For example “illicit drug use” and ”blood” occurred in 18.3 and 1.2 times more MIMIC records respectively. In the context of the MIMIC records (taken from adult ICU patients) these phrases are not relevant to NAT, but in the training of our models on NAT-specific records, these words were designated as indicative of positive NAT. This disparity presents a potential reason why the rules-based model has a high false positive rate when tested on the MIMIC database, and highlights the context dependence of the models presented in this paper.
