# Supplementary figures and images for: A natural language processing and deep learning approach to identify child abuse from pediatric electronic medical records
Source: PLoS One. 2021 Feb 26;16(2):e0247404. doi: 10.1371/journal.pone.0247404 (PMC7909689; doi:10.1371/journal.pone.0247404)

**S1 Table. Architecture and Hyperparameters for the chosen model in each strategy**
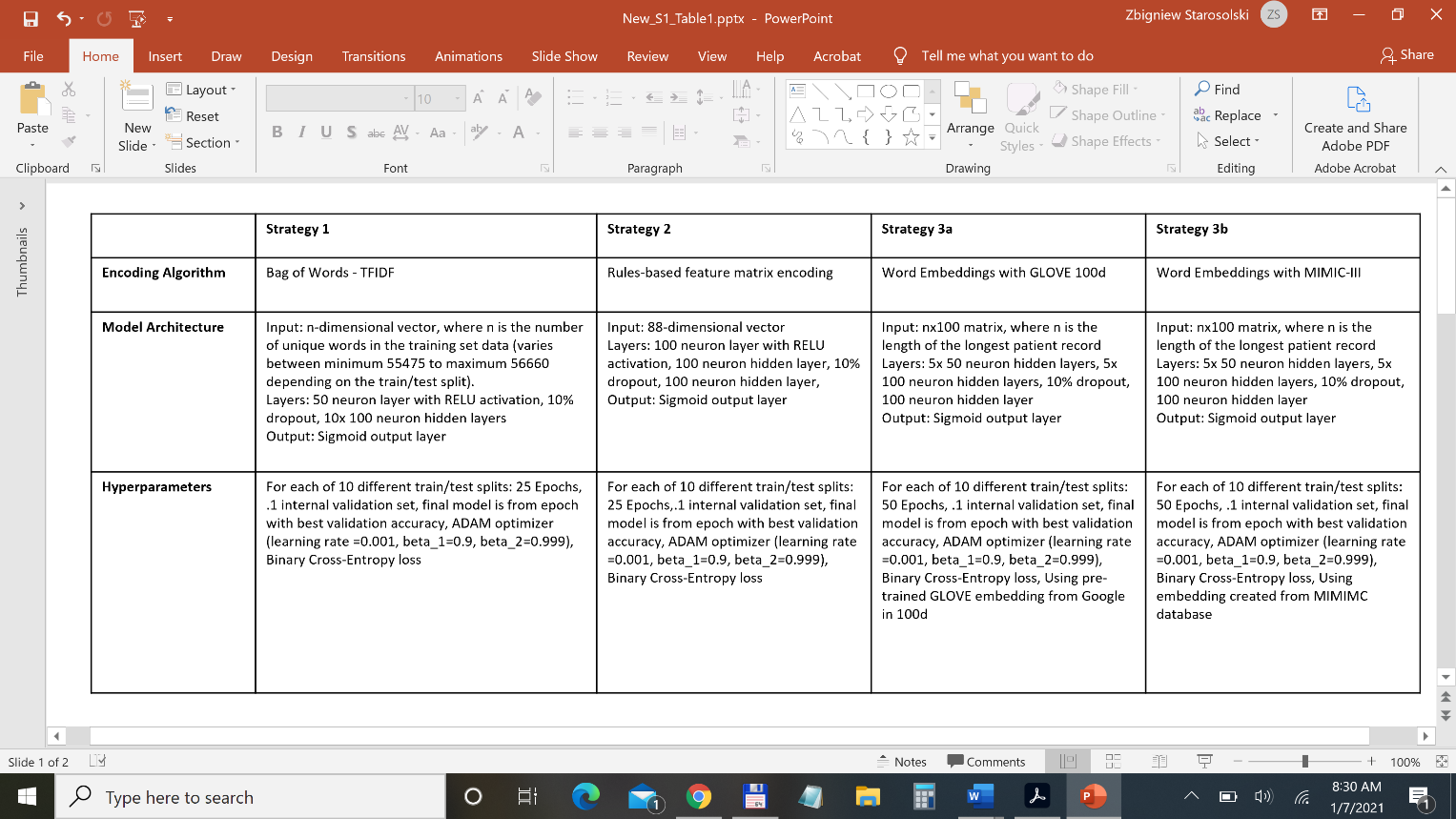

Supplement: S1 Table — (DOCX) [file pone.0247404.s008.docx]
