# Supplementary material for: A natural language processing and deep learning approach to identify child abuse from pediatric electronic medical records
Source: PLoS One. 2021 Feb 26;16(2):e0247404. doi: 10.1371/journal.pone.0247404 (PMC7909689; doi:10.1371/journal.pone.0247404)
Supplement: S2 Table — (DOCX) [file pone.0247404.s009.docx]

**S2 Table. Comparison of Logistic Regression vs. MLP performance with BOW and RB encodings**
